# Evolution of Guideline Recommendations on Insulin Therapy in Type 2 Diabetes Mellitus Over the Last Two Decades: A Narrative Review

**DOI:** 10.2174/1573399819666230116150205

**Published:** 2023-08-02

**Authors:** Manoj Chadha, Sunil M. Jain, Rajeev Chawla, Mala Dharmalingam, Tirthankar Chaudhury, P.G. Talwalkar, Sudhir Tripathi, S.K. Singh, Manish Gutch, Arundhati Dasgupta

**Affiliations:** 1 Department of Endocrinology, P.D. Hinduja Hospital, Mumbai, Maharashtra, India;; 2 TOTALL Diabetes Hormone Institute, Indore, Madhya Pradesh, India;; 3 Department of Endocrinology, North Delhi Diabetes Centre, Rohini, New Delhi, India;; 4 Department of Endocrinology, MSR Medical College & Hospital, Bangalore, Karnataka, India;; 5 Department of Endocrinology, Apollo Gleneagles Hospital, Kolkata, India;; 6 Talwalkar Diabetes Clinic, Mumbai, Maharashtra, India;; 7 Department of Endocrinology & Metabolism, Sir Gangaram Hospital, New Delhi, India;; 8 Department of Endocrinology, Institute of Medical Sciences, BHU, Varanasi, Uttar Pradesh, India;; 9 Department of Endocrinology and Diabetes, Medanta Hospital, Lucknow, Uttar Pradesh, India;; 10 Department of Endocrinology, Rudraksh Superspeciality Care, Siliguri, West Bengal, India

**Keywords:** Guidelines, insulin, type 2 diabetes mellitus, pharmacotherapy, oral antidiabetics, hemoglobin levels

## Abstract

The prevalence of type 2 diabetes mellitus has been increasing worldwide. As the therapeutic options for type 2 diabetes mellitus have evolved over the last 2 decades, national and global guidelines related to type 2 diabetes mellitus pharmacotherapy issued by various organizations have tended to vary in their recommendations. This narrative review aimed to analyze the key recommendations by major global and national guidelines on the initiation of insulin therapy in patients with type 2 diabetes mellitus over the last 20 years. Strategies for insulin therapy for titration and intensification were also assessed. All guidelines recommend initiation of insulin (basal/premixed/other formulations) when glycemic targets are not achieved despite lifestyle measures and oral antidiabetic drugs. In the recent decade, early initiation of insulin has been recommended when the glycated hemoglobin levels are >10% or blood glucose levels are ≥300 mg/dL (16.7 mmol/L). Initiation is recommended at a dose of 10 units or 0.1-0.2 U/kg. Titration is advised to achieve the optimal dosage, while intensification is recommended when glycemic targets are not achieved despite titrating to an acceptable level. Glucose monitoring at periodic intervals is recommended for adequate glycemic control. The guidelines further suggest that the choice of insulin should be individualized, considering the clinical status of patients with type 2 diabetes mellitus. The physicians as well as patients should be a part of the decisions made regarding the therapeutic choice of regimen, preparation, and delivery device.

## 1 INTRODUCTION

Globally, type 2 diabetes mellitus was diagnosed in 537 million adults aged 20-79 years in 2021 and is projected to affect 783 million adults by 2045 [1]. China, India, and the United States of America were among the top three countries with 140 million, 74 million, and 32 million adults with type 2 diabetes mellitus in 2021, respectively [1]. A systematic review of 16 guidelines and 328 statements highlighted that approximately 77%-100% of the published statements and 95% of the guidelines endorsed the benefit of tight glycemic control in type 2 diabetes mellitus patients [2]. Several landmark randomized clinical trials (RCTs) have emphasized the role of insulin therapy in achieving tight glycemic control and its subsequent benefits in reducing the likelihood of developing microvascular and macrovascular complications [3]. It has been estimated that 150-200 million individuals require insulin therapy globally [4]. Side effects such as hypoglycemia and weight gain, along with insulin distress, have often impeded the adoption and reach of insulin to patients. However, the availability of insulin analogs has improved the effectiveness of insulin therapy in addition to minimizing the risk of associated hypoglycemia [4].

Both global and national guidelines have evolved over the years with recommendations for timely insulin initiation in type 2 diabetes mellitus. The therapeutic options in the management of type 2 diabetes mellitus have also widened with the development of new oral drugs and injectables [5]. As a result, there is a need to compare and analyze the major guidelines to understand the changes in the recommendations on insulin therapy over the last two decades. This review article aims to analyze the major guidelines and highlight the available clinical evidence supporting the changes in the recommendations in those guidelines over the last two decades. This would be one of the few comprehensive narrative reviews to summarize the recommendations on insulin therapy from all major global and national guidelines, and will help guide physicians in choosing the most appropriate regimen for their patients.

## 2 MATERIALS AND METHODS

A comprehensive literature search was carried out in MEDLINE-PubMed and Google Scholar to identify relevant guidelines related to pharmacological/medical management of type 2 diabetes mellitus, which were published from January 2001 till date. The key guidelines that were planned to be reviewed included those from the Research Society for the Study of Diabetes in India (RSSDI), Endocrine Society of India (ESI), American Diabetic Association (ADA), American Association of Clinical Endocrinology (AACE), World Health Organization (WHO), International Diabetes Federation (IDF), National Institute for Health and Clinical Excellence (NICE), Diabetes Canada, Royal Australian College of General Practitioners (RACGP), Society for Endocrinology, Metabolism, and Diabetes of South Africa (SEMDSA), and Malaysian Clinical Practice Guideline. Guidelines from global organizations were chosen as they are recognized as global standards in type 2 diabetes mellitus management and are updated periodically based on the availability of newer evidence and therapeutic innovations. Keeping geographical variations in mind, prominent regional guidelines from Canada, Australia, India, South Africa, and Malaysia were also included due to their recognition as standards for type 2 diabetes mellitus management within the respective regions. The primary objective was to analyze the key recommendations by major global and national guidelines on the initiation of insulin therapy in patients with type 2 diabetes mellitus. The secondary objective was to review the guidelines to understand the strategies for insulin therapy for titration and intensification.

## 3 RESULTS

Guidelines and consensus statements from 13 global and regional organizations published between 2001 and 2021 were identified during the initial search (Table **1**). These were segregated based on the publishing organization and year of publication and evaluated further for statements/recommendations related to:

• Insulin initiation: timing of insulin initiation, number of oral antidiabetics to be used before initiating insulin, other patient-centric criteria for insulin initiation, insulin regimen (basal insulin or premixed/other insulin regimens), and dose of insulin recommended at the initiation.

• Insulin dose titration: frequency of dose titration, parameters to be considered for the dose titration, dose-titration regimen, and maximum amount of dose to be titrated before further intensification of insulin therapy.

• Insulin intensification: timing of intensifying insulin therapy and insulin regimen (by addition of prandial insulin or switching to premixed/coformulation).

Initiation of insulin therapy is recommended when the glycemic goal is not maintained with combination therapy of 2-3 prior lines of oral antidiabetic (OAD) medications at their optimized doses. Insulin as first-line therapy has been recommended if the glycated hemoglobulin level ≥7.5%-9.0% and symptomatic hyperglycemia are noted. The majority of the global guidelines, including the ADA, IDF, and AACE recommend initiation with basal insulin [6-8]. On the contrary, the NICE, RACGP, and RSSDI recommend initiation with either basal or premixed insulin [9-12]. The dose recommended for basal insulin at initiation can either be 10 units or may vary according to weight and glycated hemoglobulin values.

For insulin dose titration, most guidelines recommend titrating at a rate of 2-3 units of insulin every 3 days, few of the guidelines have recommended titrating in terms of percentage (5%-10% or 10%-15%) of the current dose. There have also been recommendations rarely to titrate the insulin dose at the rate of 1 U every day. Self-titration regimen is mostly recommended. While intensifying insulin therapy, the majority of guidelines recommend the addition of prandial insulin before the major meal followed by further addition of prandial insulin before other meals as required. However, few guidelines also recommend intensifying insulin therapy by switching to a premixed regimen. The basal-bolus regimen is recommended if the switch to a premixed regimen does not help achieve target glycemic levels. These are enumerated in Tables **2**-**4**.

## 4 DISCUSSION

### 4.1 Recommendations on the Timing of Initiating Insulin

Insulin therapy was traditionally postponed for prolonged periods and lifestyle and a combination of oral agents was preferred to achieve glycemic control. However, over the last decade, this has been revised and early initiation of insulin therapy (primarily basal insulin therapy) in combination with oral agents has been recommended [40]. While the guidelines mention that therapeutic choices for each patient are distinct and determined by the patient’s clinical status, insulin has been universally recommended as a third-or fourth-line therapy when adequate doses of two to three noninsulin agents administered for 3-6 months fail to achieve glycated hemoglobulin targets, or if organ dysfunction contraindicates the use of oral agents [12, 60]. Insulin therapy is also recommended among individuals with glycated hemoglobulin ≥ 7.5% despite two or three oral antidiabetics [48]. Nevertheless, early introduction of insulin is suggested if there is evidence of ongoing catabolism (weight loss), if symptoms of hyperglycemia are present, or when glycated hemoglobulin levels (>10%) or blood glucose levels (≥300 mg/dL) are very high [37, 38, 61, 63]. Insulin initiation has been recommended as the first-line approach in treatment naïve individuals if markedly symptomatic and/or elevated blood glucose levels (>250-300 mg/dL) or glycated hemoglobulin levels (>10%) [29, 39]. A short course of insulin for about a month is also recommended if there is evidence of glucose toxicity and lipotoxicity at the time of diagnosis [13].

### 4.2 Recommendations on the Number of Oral Antidiabetics to be used before Initiating Insulin

According to the AACE/ACE 2020 [8] recommendation, dual or triple therapy should be tried in cases where glycated hemoglobulin level is >9.0% and patients are asymptomatic before initiating insulin; insulin can, however, be initiated in symptomatic patients with/without other agents. Dual therapy for 3 months followed by triple therapy (if dual therapy is not successful) for another 3 months should be tried in patients with glycated hemoglobulin levels ≥7.5%-9.0%, before initiating insulin [8]. According to the ADA 2020 and 2021 guidelines, metformin can be combined with any of the six treatment options (sulfonylurea, thiazolidinedione, DPP-4 inhibitor, SGLT2 inhibitor, GLP-1 RA, or basal insulin) if the glycated hemoglobulin targets are not achieved within 3 months of metformin initiation [6, 38]. Basal insulin is considered an ideal option when glycated hemoglobulin targets are not achieved with dual therapy [42, 60]. Insulin initiation is also recommended in treatment naïve individuals if they have symptomatic hyperglycemia and the glycated hemoglobulin levels are >9%. In the case of individuals with glycated hemoglobulin levels ≥ 7% and <9%, basal insulin is suggested as a part of dual therapy or triple therapy [42].

According to the RSSDI consensus statements, there are several newer and more effective oral antidiabetics available, however, insulin should be considered in cases where the patient fails to achieve or maintain glycated hemoglobulin levels after administration of three oral antidiabetics; moreover, one of these should be a new agent. Insulin should also be considered if the patient is intolerant to any individual agent or combination of agents [13]. Insulin initiation may also be justified if organ dysfunction contraindicates the use of oral agents [12]. Australian guidelines suggest considering a GLP-1RA before commencing insulin unless a person has extreme hyperglycemic symptoms or a glycated hemoglobulin level of >11% [54]. Additionally, insulin should never be delayed if glycated hemoglobulin remains high despite the use of newer oral agents as their glucose-lowering potential is relatively less compared to insulin [13].

### 4.3 Recommendations on the Choice of Initial Insulin

#### 4.3.1 Basal Insulin

Basal insulin has been recommended by the AACE/ACE as a second-line alternative in patients with glycated hemoglobulin ≥7.5%-9.0%, if dual therapy or triple therapy with oral antidiabetics does not help achieve the glycemic target within a period of 3 months [8]. This recommendation has remained unchanged between 2015 and 2020. The ADA, however, recommends the initiation of basal insulin among patients with glycated hemoglobulin >7% [6]. This has been consistently mentioned across all the ADA guidelines published between 2006 and 2021 [17-38]. Furthermore, the ADA suggests basal insulin alone as the most convenient initial insulin regimen [38]. The AACE guidelines also support the initiation of insulin with long-acting basal insulin in the majority of cases [42]. The NICE guidelines recommend initiation with human neutral protamine Hagedorn (NPH) insulin, whereas long-acting basal insulin has been suggested for patients with a higher risk of developing hypoglycemia, and in circumstances where twice-daily injection may be burdensome [46]. According to the Royal Australian College of General Practitioners (RACGP) guidelines, basal insulin has a lesser risk of hypoglycemia and should be preferred among patients with consistently high fasting blood glucose (FBG) levels [54].

#### 4.3.2 Premixed/other Insulin Regimens

According to the NICE and RACGP guidelines, initiation with premixed or coformulated insulin may be more appropriate and simpler among patients with constantly elevated fasting as well as postprandial glucose [48, 54]. The AACE guidelines suggested premix insulin as an option for insulin initiation between 2006 and 2010 [39, 41]. However, the preference was changed to basal insulin instead of premix in guidelines published after 2011. This was attributed to the reduced dosage flexibility and increased risk of hypoglycemia with premixed insulin compared to basal or basal-bolus regimens [42].

### 4.4 Dose of Insulin Recommended at Initiation

Basal insulin therapy has been recommended universally to be initiated at a dose of 10 units (U) or 0.1-0.2 U/kg. A higher dosage (0.3-0.4 U/kg/day) is considered reasonable for patients with severe hyperglycemia. [31] According to the AACE guidelines, basal insulin should be initiated at a dose of 0.1-0.2 U/kg among those with glycated hemoglobulin <8%, whereas 0.2-0.3 U/kg is suggested for those with glycated hemoglobulin >8% [8]. According to the RSSDI-ESI guidelines, basal insulin is to be initiated at a dose of 0.1-0.2 U/kg if glycated hemoglobulin is <8%, whereas a dose of 0.2-0.3 U/kg is preferred if glycated hemoglobulin >8% [12]. Premixed or coformulated insulin can be initiated at a low dose of 10 units or 0.1-0.2 U/kg and titrated as needed [54]. It can be given as a once-daily injection at the main meal [13, 15].

### 4.5 Recommendations on Dose Titration

Self-titration regimen with an increase in the dose of two basal insulin units every 3 days or with biweekly or more frequent contact with a healthcare professional has been recommended by the IDF [58]. Larger increments for basal insulin of up to 4 units every 3 days can be followed if fasting glucose is >180 mg/dL [21-27]. The ADA recommendation about the dose titration has varied between 2018 and 2021. While according to the recommendations until 2018 [36], advancement to combination injectable therapy was recommended if the basal insulin dose was >0.5 U/kg/day, it was modified to >0.7-1.0 U/kg in the 2019 guidelines [37]. However, in the 2020 guidelines, the upper limit of basal insulin has been modified to >0.5 U/kg/day [6]. The guidelines caution about the possibility of overbasalization beyond this dose, which is associated with an increased risk of hypoglycemia [6, 38]. The dosage of premixed insulin can be titrated twice daily to achieve the FPG target of <110 mg/dL. The once-daily dose can be split into equal breakfast and dinner doses (50-50) [13].

### 4.6 Recommendations on Insulin Intensification

Glucose monitoring and adjustment of medications at periodic intervals are recommended if intensification of insulin therapy is planned. Basal, along with prandial insulin analogs, is preferred over NPH and regular insulin or premixed insulin combination according to the AACE guidelines. Basal-bolus insulin regimens are recommended for intensive insulin therapy owing to dosage flexibility [42]. The ADA algorithm for combined injectable therapy in type 2 diabetes mellitus changed in 2017, in which basal insulin along with GLP-1 RA was preferred compared to basal plus rapid-acting insulin or two daily doses of premix insulin [35].

According to the RSSDI-ESI recommendations, basal along with prandial insulin before the meal that is associated with the largest PPG excursion is recommended as a part of intensification for individuals on basal regimen. This approach offers flexibility and can be further intensified if necessary (for covering 2 or 3 meals). The basal-bolus regimen is considered the most effective and physiological regimen for intensification. However, it requires frequent monitoring [12]. The ADA 2021 guidelines recommend intensification by adding prandial to basal insulin. The addition of premix insulin (twice daily) is suggested if glycated hemoglobulin levels remain high despite adding prandial insulin [38].

According to the 2021 ADA guidelines, the addition of prandial insulin (4 IU/day or 10% of basal dose) has been recommended if glycated hemoglobulin remains above target levels despite the addition of basal insulin to GLP-1 RAs. The dose of prandial insulin can be increased by 1-2 IU/day or 10%-15% per week. Stepwise additional injections of prandial insulin have been suggested if the glycated hemoglobulin target is not achieved. Alternatively, switching over to two or three doses of premixed insulin has been suggested in patients on basal insulin in whom additional prandial coverage is desired [38]. However, reduced dosage flexibility and increased risk of hypoglycemia are of concern with premixed insulin [42]. Intensification of premixed insulin can be carried out by splitting the once-daily dose into twice daily or thrice daily injections if the FPG is >110 mg/dL. The addition of 2-6 U or 10% of the total daily premix dose before lunch is suggested. The dosage can be split thrice a day if further intensification is desired [13].

### 4.7 Recommendation on Monitoring Glycemic Control with Insulin

Self-monitored blood glucose (SMBG) has been recommended for monitoring glycemic control by various guidelines [12, 15, 17, 54]. Structured SMBG, along with appropriate therapeutic interventions, has been suggested to be associated with greater HbA1c reduction compared to approaches without structured SMBG [12]. The guidelines also acknowledge the role of continuous glucose monitoring (CGM) in the improved management of type 2 diabetes mellitus and have predicted significant usage of CGM in the future [12, 15, 38].

### 4.8 Note on Hypoglycemia Prevention

The risk of hypoglycemia is one of the major concerns that limit the usage of insulin both among practitioners as well as patients. While the majority of the recommendations do recommend periodic monitoring of HbA1c levels to ensure optimal control of blood glucose levels, identification of an optimal level for a particular population is challenging owing to individual differences in ethnicity, dietary practices, lifestyle, hypoglycemia risk, and other adverse effects. Therefore, counseling about the risk of hypoglycemia and steps to recognize, prevent, and treat hypoglycemia has been recommended for all patients for whom initiation of insulin is planned. Adequate guidance about SMBG, CGM, dose adjustments, storage, and administration should also be provided to all patients and caregivers.

## 5 LIMITATIONS

The current review article includes only a limited number of globally established guidelines considered as global standards in type 2 diabetes mellitus management and a few regional guidelines that are accepted within the respective regions. The guidelines considered in the current review article may, however, not entirely reflect the insulin usage protocols/practices followed across all countries and individual institutions/hospitals across the globe in the management of type 2 diabetes mellitus. Nevertheless, an attempt has been made to capture all guidelines that are relevant to most of the geographical regions of the world.

## CONCLUSION

Initiation of insulin (basal/premixed/other formulations) has been universally recommended if glycemic targets are not achieved despite lifestyle measures and oral antidiabetics. Notably, early initiation of insulin has been recommended in the recent decade when the glycated hemoglobulin levels are >7% despite optimization of 2-3 prior lines of oral antidiabetics. The choice of insulin, dosage, titration, and intensification is influenced by the clinical status of the patients and needs to be individualized. Therapy with basal insulin is recommended in view of its ease and convenience of initiation, flexible-dose titration regimens, and low risk of hypoglycemia, more so in patients with elevated fasting glucose. Furthermore, basal insulin intensification *via* a stepwise addition of prandial insulin is a more physiological and flexible intensification strategy. Premix/coformulation insulin can be used in patients having both fasting and postprandial hyperglycemia together and also to reduce the number of injections at the time of intensification. Healthcare providers should emphasize the importance and utility of insulin in maintaining glycemic control. Educating and empowering patients is an important step in improving the acceptability of insulin. Furthermore, physicians as well as patients should be part of decisions regarding the therapeutic choice of insulin regimen, preparation, and delivery device.

## DISCLOSURE

All authors had full access to the articles reviewed in this manuscript, have read and reviewed the final draft of this manuscript, and take complete responsibility for the integrity and accuracy of this manuscript. The content published herein solely represents the views and opinions of the authors. The details published herein are intended for informational, educational, academic, and/or research purposes and are not intended to substitute for professional medical advice, diagnosis, or treatment.

## Figures and Tables

**Table 1:** Details of the guidelines considered for evaluation.

**Organization**	Year*	**Total**
ADA guideline	2002-2021	20
ADA EASD consensus statement	2006, 2009	2
ADA EASD position statement	2012, 2019	2
AACE guidelines	2007, 2011	2
AACE/ACE consensus statement	2009, 2015-2018, 2020	6
Malaysian clinical practice guideline	2009, 2015, 2020	3
NICE guidelines	2008, 2009, 2015, 2019, 2020	5
SEMDSA guidelines	2009, 2012, 2017	3
WHO guidelines	2004, 2006, 2018, 2020	4
IDF guidelines	2011-2013, 2017	4
RSSDI	2017, 2019	2
RSSDI-ESI clinical practice guidelines	2020	1
Diabetes Canada	2018, 2020	2
RACGP guidelines	2011, 2012, 2014, 2016-2018, 2020	4
GRAND TOTAL	-	60

**Note:** *Includes guidelines that were available online.

**Abbreviations:** ADA: American Diabetes Association; AACE: American Association of Clinical Endocrinology; ACE: American College of Endocrinology; EASD: European Association for the Study of Diabetes; ESI: Endocrine Society of India; IDF: International Diabetes Federation; NICE: National Institute for Health and Care Excellence; RACGP: Royal Australian College of General Practitioners; RSSDI: Research Society for the Study of Diabetes in India; SEMDSA: Society for Endocrinology, Metabolism, and Diabetes of South Africa; WHO: World Health Organization.

**Table 2:** Asian guideline recommendations regarding basal and premix insulin for type 2 diabetes mellitus.

Recommendations*	**RSSDI 2017-2020 [** **11** **-** **13** **]**	**Malaysian 2009 [** **14** **]**	**Malaysian 2015 [** **15** **]**	**Malaysian 2020 [** **16** **]**
Timing of insulin initiation	If a study of adequate doses of two to three noninsulin agents for 3-6 months fails to achieve HbA1c targets, or if organ dysfunction contraindicates the use of oral agents, the addition of insulin may be justified	Patients who are not reaching targets (HbA1c <6.5%) after 3-6 months on optimal doses of combination therapy [*Grade C*]	If targets are not met after optimal OAD therapy, consider adding GLP-1 RA (if HbA1c<10.0%) or basal insulin *[Grade A]*	Initiate when diabetes is not adequately controlled on maximum OGLDs ± GLP1-RA. As initial therapy in newly diagnosed T2DM, • in the presence of symptomatic hyperglycemia and evidence of ongoing catabolism, *[Level III]* • when HbA1c >10% or FPG >13.0 mmol/L
Number of OADs to be used before initiating insulin	Two to three	Not specified; mentioned as optimal doses of combination therapy	Not specified; mentioned as optimal doses of combination therapy	Not specified; mentioned as maximum number of oral agents
Choice of initial insulin	Insulin initiation with either a basal or premixed/coformulation insulin	Types of insulin regimes • OAD agents + basal insulin or premixed insulin once a day • Metformin + premixed insulin more than once a day • Metformin + basal insulin + prandial insulin	If targets have not been reached after optimal OAD therapy, consider adding: • Prebed basal insulin, or • Pre-dinner premixed insulin, or • GLP-1 RA, as an alternative to intermediate or long-acting insulin with less incidence of • Hypoglycemia and weight gain (provided the HbA1c is <10.0%) *[Level I]*	Options include: • Basal insulin *[Level I]* OR • Premixed insulin once or twice daily
Dose of insulin at initiation	Basal insulin: Initiate with 10 U at bedtime and check FBSL Premixed insulin: Initiate with 10-12 U/day or 0.1-0.2 U/kg/day	Insulin can be initiated at 10 U a day or 0.1-0.2 U/kg/day, • set FPG target and choose evidence-based titration	Insulin can be initiated at 10 U a day or 0.1-0.2 U/kg/day, • set FPG target and choose evidence-based titration	Insulin can be initiated at 10 U a day or 0.1-0.2 U/kg/day *[Level I]* • set FPG target and choose evidence-based titration
Dose titration	Basal insulin: Increase dose by 1 U/day or 3 U every 3 days by patient self-titration till target FBSL is achieved Premix insulin: Split once-daily regimen to twice-daily regimen (50:50)	Dose to be increased every third or fourth day by 2-4 units until target FPG is achieved	Dose to be increased every third or fourth day by 2-4 units until target FPG is achieved	Dose to be increased every third or fourth day by 2-4 units until target FPG is achieved
Dose intensification	Basal insulin: • Add prandial insulin before the largest meal • Add GLP1-RA or SGLT2i or DPP4i • Switch to premix insulin twice daily Premix insulin: Add 2-6 U or 10% of the total daily dose before lunch	Add prandial insulin with the biggest meal or add premix insulin at breakfast, if target HbA1c is not achieved in 3-6 months	Add prandial insulin with the biggest meal or add premix insulin at breakfast, if target HbA1c is not achieved in 3-6 months	Basal insulin: Add prandial insulin 4 U/meal or 10% of basal dose or convert to twice daily premixed insulin Premix insulin: Uptitrate to twice daily or use coformulation twice daily

**Note:** *Grade of evidence has been specified in italics wherever available.

**Abbreviations:** DPP4i: Dipeptidyl peptidase-4 inhibitor; FBSL: Fasting blood sugar level; FPG: Fasting plasma glucose; GLP1-RA: Glucagon-like peptide-1 receptor agonists; OAD: Oral antidiabetic drug; OGLDs: Oral glucose-lowering drugs; RSSDI: Research Society for the Study of Diabetes in India; SGLT2i: Sodium/glucose cotransporter-2 inhibitor; T2DM: Type 2 diabetes mellitus.

**Table 3:** American guidelines* regarding basal and premix insulin for type 2 diabetes mellitus.

**Recommendations**	**ADA 2001-2010ᵃ [** **17** **-** **27** **]**	**ADA 2011-2015^b^ [** **28** **-** **33** **]**	**ADA 2016-2020 [** **34** **-** **38** **]**	**ADA - 2021 [** **39** **]**	**AACE 2001-2020 ** **[** **40** **, ** **41** **]**	**AACE/ACE 2009 - 2020 [** **42** **-** **46** **]**
Timing of insulin initiation	Early initiation of insulin would be a safer approach for individuals presenting with weight loss, more severe symptoms, and glucose values >250-300 mg/dl	In patients with newly diagnosed type 2 diabetes and markedly symptomatic and/or elevated blood glucose levels or HbA1c, consider initiating insulin therapy (with or without additional agents)	The early introduction of insulin should be considered if there is evidence of ongoing catabolism (weight loss), if symptoms of hyperglycemia are present, or when HbA1c levels (>10% [86 mmol/mol]) or blood glucose levels (≥300 mg/dL [16.7 mmol/L]) are very high	The early introduction of insulin should be considered if there is evidence of ongoing catabolism (weight loss), if symptoms of hyperglycemia are present, or when HbA1c levels (>10% [86 mmol/mol]) or blood glucose levels (≥300 mg/dL [16.7 mmol/L]) are very high	For HbA1c level >9.0% and asymptomatic - try dual or triple therapy before initiating insulin; for symptomatic patients initiate insulin with/without other agents. For HbA1c level ≥7.5% to 9.0% - try dual therapy (if dual therapy is not useful) for 3 months followed by triple therapy for 3 months before initiating insulin	Insulin to be initiated among patients with HbA1c level >8.0% uncontrolled with dual or triple oral agents
Number of OADs to be used before initiating insulin	Although three oral agents can be used, initiation and intensification of insulin therapy is preferred based on effectiveness and expense	If the HbA1c target is not achieved after 3months of metformin monotherapy, consider one of the five treatment options combined with metformin: a sulfonylurea, TZD, DPP-4 inhibitor, GLP-1 receptor agonist, or basal insulin	If noninsulin monotherapy at maximum tolerated dose does not achieve or maintain the HbA1c target after 3 months, add a second oral agent, a glucagon-like peptide 1 receptor agonist, or basal insulin	If the HbA1c target is not achieved after approximately 3 months, metformin can be combined with any one of the preferred six treatment options: sulfonylurea, thiazolidinedione, DPP-4 inhibitor, SGLT2 inhibitor,GLP-1 RA, or basal insulin; the choice of which agent to add is based on drug-specific effects and patient factors	For HbA1c level >9.0% and asymptomatic - try dual or triple therapy before initiating insulin; for symptomatic patients initiate insulin with/without other agents. For HbA1c level ≥7.5% to 9.0% - try dual therapy (if dual therapy is not useful)for 3 months followed by triple therapy for 3 months before initiating insulin	2 or 3
Choice of initial insulin	-	Basal insulin considered as the most convenient regimen	Basal insulin considered as the most convenient regimen	Basal insulin considered as the most convenient regimen	Basal, basal-bolus, prandial, or premixed regimen	Preference changed from basal, basal-bolus, prandial, or premixed regimen in 2009 to basal insulin from 2016 onwards
*Basal Insulin*	-	-	-	-	Long-acting basal insulin is the initial choice for initiation of insulin therapy	-
*Premixed/other insulin regimens*	No specific recommendations	Twice daily premix insulin suggested as an option with less flexible dosing (2012 ADA EASD guideline)	-	-	Premix preferred in patients with adherence issues	-
Dose of insulin at initiation	10 units or 0.2 U/kg	10 units or 0.2 U/kg	10 units per day or 0.1-0.2 units/kg/day, depending on the degree of hyperglycemia	10 units per day or 0.1-0.2 units/kg/day, depending on the degree of hyperglycemia	Basal insulin: 0.1-0.2 U/kg/day if HbA1c <8% 0.2-0.3 U/kg/day if HbA1c >8% Premix insulin: Administered at the largest meal once daily or at the 2 largest meals twice daily	Basal insulin: 0.1-0.2 U/kg if HbA1c <8%; 0.2-0.3 U/kg if HbA1c is >8%
Dose titration	Increase daily by 2 units every 3 days till the glycemic goals are achieved	Adjust by 10-15% or 2-4 U once-twice weekly	Adjust by 10-15% or 2-4 U once-twice weekly	Increase 2 U every 3 days	Titrate 2 U/day every 2-3 days until glycemic goals are reached	Insulin titration every 2-3 days by 2 U to reach glycemic goal
Dose intensification	Larger increments - 4 units every 3 days, can be followed if fasting glucose is >180 mg/dL	Add 1 rapid insulin injection before meal; if not controlled, consider basal-bolus Alternatively, change to premix insulin twice daily	Add 1 rapid insulin injection before meal; if not controlled, consider basal-bolus Alternatively, change to premix insulin twice daily	Add prandial insulin at 4 IU/day or 10% of the basal insulin If HbA1c above target, consider stepwise addition of prandial insulin OR self-mixed or split insulin regimen OR twice daily premix insulin regimen	Prandial therapy with GLP-1 receptor agonist, SGLT2 inhibitor, DPP-4 inhibitor or prandial insulin (0.3-0.5 U/kg)	Consider adding GLP1-RA/SGLT2i/DPP4i OR prandial insulin for intensification

Note: *Grade of evidence has been specified in italics wherever available. ^a^Includes ADA consensus statements for 2006 and 2009; ^b^Includes ADA-EASD 2012 guideline.

ADA: American Diabetes Association; AACE: American Association of Clinical Endocrinology; ACE: American College of Endocrinology; DPP4i: Dipeptidyl peptidase-4 inhibitor; FPG: Fasting plasma glucose; GLP1-RA: Glucagon-like peptide-1 receptor agonists; OAD: Oral antidiabetic drug; SGLT2i: Sodium/glucose cotransporter-2 inhibitor; TZD: Thiazolidinediones.

**Table 4:** Other guidelines regarding basal and premix insulin for type 2 diabetes mellitus.

Recommendations*	**NICE 2008-2020 ** **[** **46** **-** **50** **]**	**RACGP 2011-2012 [** **51** **], 2014 [** **52** **], 2016-2018 [** **53** **], 2020 [** **54** **]**	**Diabetes Canada (Clinical Practice Guidelines) 2018 [** **55** **]**	**Diabetes Canada (Clinical Practice Guidelines) 2020 [** **56** **]**	**IDF 2011-2017 ** **[** **57** **-** **60** **]**	**SEMDSA 2009-2017 [** **61** **-** **63** **]**
Timing of insulin initiation	Nothing specific; however, insulin is recommended following inability to control blood glucose with OADs (mono- or combination)	International and Australian guidelines suggest considering a GLP-1 RA before commencing insulin, unless a person has extreme hyperglycemic symptoms or an HbA1c of >11%. Insulin should be initiated in patients with type 2 diabetes mellitus who are taking maximal doses of non-insulin glucose-lowering medicines and who have suboptimal glycemic control (HbA1c or blood glucose above individualized target), whether they are asymptomatic or symptomatic	If glycemic targets are not achieved with existing antihyperglycemic medication(s), other classes of agents should be added to improve glycemic control. *[Grade B]* Insulin should be immediately initiated in people with evidence of metabolic decompensation (such as marked hyperglycemia, ketosis, or unintentional weight loss) and/or symptomatic hyperglycemia, regardless of HbA1c level	In people not achieving glycemic targets on existing noninsulin antihyperglycemic medication(s) *[Grade B].*	Third-line therapy When glucose control targets are no longer being achieved, start insulin, or add a third oral agent. If starting insulin, add basal insulin or use premix insulin	Consider insulin as first-line therapy at diagnosis, and at any other point in the course of the disease, in the setting of metabolic decompensation with any of the following features: • Catabolism (marked weight loss) • Fasting plasma glucose levels >14 mmol/l • Random glucose levels consistently >16.5 mmol/L • HbA1c >10% • Presence of persistent ketogenesis, ketoacidosis, or hyperosmolar nonketotic state *[Grade C]*
Number of OADs to be used before initiating insulin	Not specified	Three	Not specified. The use of OADs varies with the risk of comorbidities such as CVD	Not specified. The use of OADs varies with the risk of comorbidities such as CVD.	Insulin is recommended as third-line therapy	Consider adding basal insulin as the third glucose-lowering drug in patients not achieving or maintaining their glycemic targets on a two-drug oral regimen, especially if targets are unlikely to be achieved with other third-line options, and there are adequate resources to support insulin initiation and titration
Choice of initial insulin	Basal insulin alone has a slightly lower risk of hypoglycemia, especially if the fasting glucose is consistently above target	Basal insulin is to be preferred if fasting glucose is consistently above target levels. Premixed or coformulated insulin may be more appropriate and simpler for a patient where fasting and postprandial glucose are both consistently elevated	In people not achieving glycemic targets on existing noninsulin antihyperglycemic medication (s), the addition of a once-daily basal insulin regimen should be considered over premixed insulin or bolus only regimens, if lower risk of hypoglycemia and/or weight gain are priorities *[Grade B]*	The addition of a basal insulin regimen should be considered over premixed insulin or bolus-only regimens, if lower risk of hypoglycemia and/or preventing weight gain are priorities *[Grade B]*	Basal insulin should be preferred, and it can be temporary	If insulin is needed at diagnosis, use either premixed insulin twice daily or basal-bolus intensive insulin therapy (specialist referral is recommended)
Dose of insulin at initiation	Start with 10 unit or 0.2 units/kg	10 units or 0.1-0.2 units/kg for premixed, coformulated, or basal insulin	Not specified. Number and timing of insulin injections may vary with the clinical situation	Not specified. Number and timing of insulin injections may vary with the clinical situation	Initiate insulin using a self-titration regimen	Initiate 10 units of basal insulin (or 0.2 U/kg) using intermediate or long-acting insulin (use insulins with a low acquisition cost; clones and biosimilar insulins are acceptable)
Dose titration	Titrate once or twice weekly at 1 to 2 units each time to achieve a target fasting blood glucose between 3.9 and 7.2 mmol/L (70 and 130 mg/dL)	Basal insulin: Adjust the dose based on previous average fasting glucose levels. Increase by 2 U/kg every 3 days until the FPG target is achieved. Premix insulin: Adjust evening dose once/twice a week based on FPB levels	Not specified. Number and timing of insulin injections may vary with the clinical situation	Not specified. Number and timing of insulin injections may vary with the clinical situation	Increase by two units every 3 days or biweekly or more frequently (as suggested by a healthcare professional)	Simple titration: Once weekly average of the last two fasting SMBG levels (use preprandial SMBG for premix or bolus insulin). Simple rapid titration: Once-daily titration according to the last fasting SMBG level (use preprandial SMBG for premix or bolus insulin). Aggressive titration: Once weekly lowest of the last 3 fasting SMBG readings (use preprandial SMBG for premix or bolus insulin)
Dose intensification	Not specified	Basal plus, basal-bolus, or switch to premix insulin. Premix insulin: Intensify to twice daily.	GLP-1 receptor agonist should, be considered before bolus insulin as add-on therapy in people on basal insulin (with or without other agents) who require antihyperglycemic treatment intensification if there are no barriers to affordability or access	Add GLP1-RA, SGLT2i, or DPP4i if target levels are not achieved. *[Grade B]* Progress to the addition of bolus insulin or multiple injections with bolus injection at each meal	-	-

**Note:** **Grade of evidence has been specified in italics wherever available.

**Abbreviations:** CVD: Cardiovascular disease; DPP4i: Dipeptidyl peptidase-4 inhibitor; FPG: Fasting plasma glucose; GLP1-RA: Glucagon-like peptide-1 receptor agonists; IDF: International Diabetes Federation; NICE: National Institute for Health and Care Excellence; OAD: Oral antidiabetic drug; RACGP: Royal Australian College of General Practitioners; SEMDSA: Society for Endocrinology, Metabolism, and Diabetes of South Africa; SMBG: Self-monitored blood glucose; SGLT2i: Sodium/glucose cotransporter-2 inhibitor.

## LIST OF ABBREVIATIONS

ADA: American Diabetic Association
AACE: American Association of Clinical Endocrinology
ACE: American College of Endocrinology
CVD: Cardiovascular Disease
DPP4i: Dipeptidyl Peptidase-4 Inhibitor
EASD: European Association for the Study of Diabetes
ESI: Endocrine Society of India
FBSL: Fasting Blood Sugar Level
FPG: Fasting Plasma Glucose
GLP1-RA: Glucagon-like Peptide-1 Receptor Agonists
IDF: International Diabetes Federation
NICE: National Institute for Health and Clinical Excellence
OAD: Oral Antidiabetic Drug
OGLDs: Oral Glucose-lowering Drugs
RACGP: Royal Australian College of General Practitioners
RCTs: Randomized Clinical Trials
RSSDI: Research Society for the Study of Diabetes in India
SEMDSA: Society for Endocrinology, Metabolism, and Diabetes of South Africa
SGLT2i: Sodium/glucose Cotransporter-2 Inhibitor
SMBG: Self-monitored Blood Glucose
T2DM: Type 2 Diabetes Mellitus
TZD: Thiazolidinediones
WHO: World Health Organization
